# Abdominal Cocoon Simulating Acute Appendicitis

**Published:** 2012-03-01

**Authors:** Kanchan Kayastha, Bilal Mirza

**Affiliations:** Department of Pediatric Surgery, The Children's Hospital and the Institute of Child Health Lahore, Pakistan

A 13-year-old girl presented to surgical emergency with pain in right iliac region for a day. The pain was localized to right iliac fossa. She also had two episodes of non-bilious vomiting. There was no previous history of such pain or vomiting, however patient gave the history of off and on constipation for the last 2 months. Her menarche started 6 months back and was uneventful and regular. She did not had any significant past medical or surgical history.

She was vitally stable. Abdomen was not distended. On palpation there was marked tenderness and guarding at right iliac region. No mass or visceromegaly was noted. Blood picture however showed normal total leukocyte count. Intravenous antibiotics and fluids started. Ultrasonography was normal. Her symptoms did not improve therefore it was decided to operate with suspicion of acute appendicitis.

At operation a mass was noted in the right lower quadrant besides a normal appendix. The incision was extended and mass delivered out. It was a pearly white thickened bowel, a foot long and just proximal to ileocecal valve (Fig. 1). Initially the nature of mass was obscured. It was just a thickened bowel. The gut proximal to the involved bowel was dilated and hypertrophied. Further exploration and manipulation of the mass revealed a pearly white membrane over the mass. The membrane was gently peeled off the mass that unveiled 3 feet small bowel being entrapped within it. The unveiled gut was of normal texture and vascularity. Mesenteric lymph nodes were not enlarged. The operative diagnosis was abdominal cocoon and about 3 feet of small bowel was packed within a pearly white tough membrane as in accordion (Fig. 2). The post operative recovery and follow up was uneventful. The histopathological examination revealed a fibrocollagenous membrane with non specific chronic inflammatory reaction. Postoperative work-up for tuberculosis was negative. 

**Figure:**
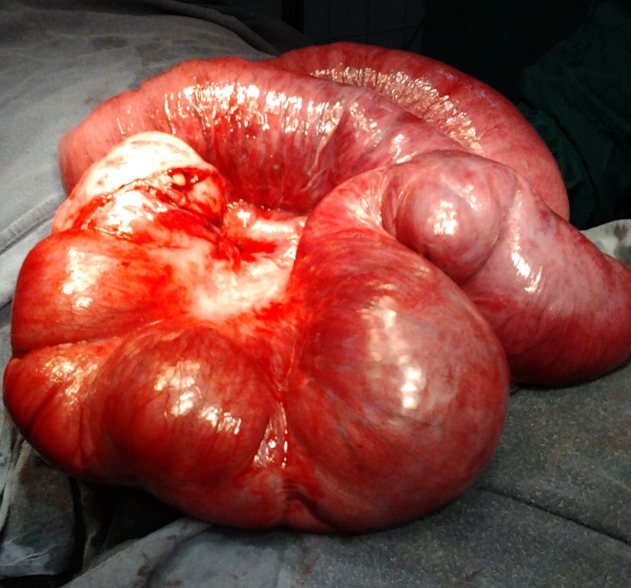
Figure 1: A feet long thick bowel mass.

**Figure:**
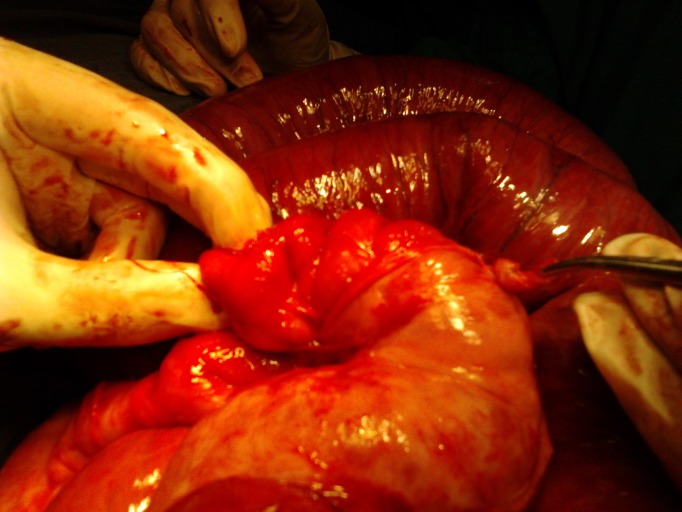
Figure 2: Unveiling of accordion like packed bowel within a pearly white membrane.

## DISCUSSION

Abdominal cocoon, also referred to as sclerosing encapsulating peritonitis, is a rare cause of acute abdomen in childhood, mostly involving young adolescent females. There is an encasement of small bowel (sometimes whole of the abdominal viscera) by a fibrocollagenous cocoon like sac which is usually formed by a nonspecific chronic inflammatory reaction. It can be idiopathic or secondary to practolol intake, chronic peritoneal dialysis, ventriculoperitoneal and peritoneovenous shunts, sarcoidosis, liver cirrhosis, leiomyomata of uterus, endometriotic cyst, tumors of ovary, abdominal tuberculosis [1,2]. In our case the cocoon was probably idiopathic in absence of features specific to other disease processes.

Abdominal cocoon usually present as sub-acute intestinal obstruction. However presentation could be chronic constipation, anorexia, weight loss and abdominal mass in rare instances. Devay et al in 2006 could find only 47 reported cases of abdominal cocoon. Out of these cases only few presented with mass in right iliac fossa [2]. In our case patient presented with pain in right iliac region and vomiting thus simulated acute appendicitis. Due to the position of the cocoon at right iliac fossa and some ongoing inflammatory process causing peritoneal irritation, the patient had sudden attack of pain in that region. 

## Footnotes

**Source of Support:** Nil

**Conflict of Interest:** None declared
